# Latent Monotonic Feature Discovery for Structural Health Monitoring

**DOI:** 10.3390/s26061898

**Published:** 2026-03-18

**Authors:** Guus Toussaint, Arno Knobbe

**Affiliations:** LIACS, Universiteit Leiden, 2333 CC Leiden, The Netherlands

**Keywords:** structural health monitoring, monotonic sensors, latent monotonic feature discovery

## Abstract

Quantifying the health of civil infrastructure using sensor data remains challenging, as degradation-related signals are typically weak and obscured by dominant environmental and operational effects. In structural health monitoring (SHM), this often results in sensor measurements that are highly periodic or intermittent, while long-term degradation manifests only as subtle drift. This study addresses the problem of extracting meaningful proxies for structural health from such data. We propose monotonicity as a guiding principle, operationalized through absolute Spearman’s rank correlation between sensor values and time. Two complementary methods are introduced. First, subgroup discovery is employed to identify structurally coherent groups of sensors that exhibit significantly elevated monotonicity, enabling the construction of robust health proxies through aggregation. Second, we present Latent Monotonic Feature Discovery (LMFD), a data-driven method inspired by equation discovery, which searches for arithmetic combinations of sensors that yield monotonic behaviour even when individual sensors are predominantly non-monotonic. The methods are evaluated on a two-year monitoring dataset from a Dutch concrete highway bridge comprising strain gauges, geophones, and temperature sensors. Results show that meaningful monotonic proxies can be derived both from naturally monotonic sensor subgroups and from composite features constructed from periodic signals. The proposed approach provides indirect yet interpretable indicators of structural health and offers a principled way to uncover latent degradation trends in long-term SHM data.

## 1. Introduction

Quantifying a structure’s health in structural health monitoring is a non-trivial task. Attaching sensors to or embedding them in a structure is a common and sensible approach for structural monitoring. Typical sensor types in this context are strain gauges [1,2], geophones [3,4], or accelerometers [1]. While such sensors are in principle sensitive to health- or degradation-related phenomena, the measured signals are often dominated by dynamic and transient effects, which obscure the subtle, gradual drift associated with long-term degradation [2,5]. Although sensor measurements themselves may be affected by environmental factors—effects that can often be mitigated [6]—a more substantial disturbance of the degradation signal arises from dynamic influences acting on the structure itself. These influences are captured by the sensor along with the signal of interest. For example, a concrete highway bridge instrumented with strain gauges will respond strongly to heavy traffic (individual trucks, congestion, etc.), but these responses are transient, and superimposed on the underlying health signal. Similarly, seasonal effects can induce large variations in the sensor readings, often drowning out traffic-related responses, not to mention the subtle drift caused by gradual degradation [1,5]. As a result, sensor measurements typically represent a mixture of signals, combining substantial periodic and intermittent components with a weak, slowly evolving trend. The central challenge in using such sensor data for structural health monitoring therefore lies in disentangling these coupled responses [7].

Our objective for this paper is to extract proxies for health, either by focusing on sensors that are more likely to capture gradual trends, or by constructing composite features from multiple sensor readings, thus producing latent features that focus on health, while attenuating the dynamic aspects of the raw sensor data. Our guiding principle for selecting or constructing proxies for health will be the *monotonicity* of a signal. A signal is deemed monotonic to the extent that it shows a consistent trend towards higher (or lower) values. This notion is operationalized by computing the absolute rank correlation between the proxy value and the time stamp, as computed through the absolute Spearman’s rank correlation ρ [8]. For a perfectly monotonic proxy—one that continuously moves in the same direction, regardless of rate—this rank correlation will be 1. Conversely, flat, periodic, or strongly intermittent signals will have a rank correlation near 0. Any signal in between, for example, when fluctuations are superimposed on a slowly drifting baseline, will have a value between 0 and 1, expressing the degree of monotonicity. Note that we compute the *absolute* rank correlation because we do not know a priori whether a particular sensor or set of sensors is expressing degradation as positive or negative influences on the measurements. We thus accept both progressively climbing and dropping signals (just not at the same time).

We should acknowledge that our proposed proxies may be of value, but do not directly measure health. For that, we would need to resort to invasive actions such as drilling cores [9], or to sensors that directly measure physical degradation processes such as corrosion or chloride ingress [10]. But in the absence of such localized, invasive, or temporary sensing, our proposed method should provide the best available alternative. Data-driven monotonic proxies should therefore be considered indirect indicators of health, pointing to potential degradation. Other than quantifying the progress, they can also help to analyse the *speed of progress*. For instance, if a proxy remains largely stable overall but exhibits pronounced, irreversible changes under specific conditions—such as extended periods of sub-zero temperatures—this behaviour may provide clues about the underlying degradation mechanisms at play. Furthermore, we acknowledge that certain degradation processes may be partially reversible (e.g., by concrete autogenous healing [1,11]), such that some structures do not exhibit completely monotonic behaviour as they age. But while the structure appears to momentarily improve according to some measurements, the underlying structural capacity continues to degrade. But even in these circumstances, monotonicity may be used as a selection mechanism to choose between informative and misleading proxies. And if one is fortunate enough to discover near-monotonic proxies—as we do in Section 4.2—those are certainly good candidates for indicators of structural health.

In some situations, one might be fortunate enough to find, amongst the applied sensors, one or more sensors that are already quite monotonic (and thus good proxies for health on their own). In such a situation, it is important to avoid premature conclusions, since it might still be the case that the monotonic process being observed is actually due to the degradation of the sensor, rather than that of the monitored structure. At face value, it is impossible to distinguish between sensor drift versus structural drift, based on an individual time series. We approach this apparent limitation from the standpoint of diversity: if a *single* sensor, or in fact all sensors of a given type, show monotonic trends, this suggests sensor degradation rather than structural change. But if, on the other hand, a diverse *group* of sensors, identified by specific physical features, shows monotonic behaviour, we can conclude that the involved sensors form valid proxies for structural health.

We approach this analysis through the machine learning method subgroup discovery [12,13], which we will employ to discover subgroups of surprisingly monotonic behaviour, identified by interpretable descriptions in terms of available meta-features. These meta-features relate to application nature of the sensor, and include properties like attachment, location, and orientation. Together, the subgroup of monotonic sensors should produce a diverse, robust collection of sensors that can be aggregated into a single proxy, for example, by computing the median of the sensors’ values. Furthermore, the uniquely identifying properties of the subgroup, expressed in terms of physical properties of the structure, not the sensor, should hint towards the nature of the degradation process that the subgroup captures.

In practice, however, the collection of available sensors will *not* show sufficient monotonicity, since the subtle drift meant to be recorded is drowned out by periodic and transient signals that are also recorded. In such situations, deriving monotonic proxies from the data is more challenging. We present a second method aimed at such challenging datasets, which we call *Latent Monotonic Feature Discovery* (LMFD) [14]. We will demonstrate how the method uses ideas from the field of equation discovery [15,16,17,18] in order to find arithmetic combinations of two or more sensors that exhibit monotonic behaviour, despite the individual signals being very periodic or intermittent. In broad terms, the composite proxies are meant to combine sensors that are sensitive to dynamic influences, in ways that these influences ‘cancel out’, while retaining the subtle drift of interest. Since it is not initially apparent which sensors include drift, and which sensors should be combined, a search process for candidate sensor combinations is executed, an approach that is common in equation discovery. Our approach performs a heuristic traversal through the search space of candidate equations, and per candidate, optimises associated parameters. Optimal proxies (arithmetic combinations of sensors with fitted parameters) are finally reported. We show how our LMFD method is able to identify highly monotonic proxies from sensors that are predominantly periodic (mostly sensitive to seasonal temperature changes).

In summary, this paper describes the following contributions:

- We propose absolute Spearman’s rank correlation between sensor values and the time stamp as a central measure for a sensor’s monotonicity. Functional combinations of sensors can be treated in the same way.
- We show how our method can reduce the likelihood of capturing sensor drift, by identifying subgroups of sensors that share structural properties, rather than sensor properties.
- We propose the application of an existing subgroup discovery method to find subgroups of sensors based on shared structural properties and significant levels of monotonicity.
- We describe how to define a median proxy for health derived from the subgroup of sensors, and how its rate of change can be correlated with meteorological features over time.
- We describe a second method for obtaining monotonic proxies, aimed at datasets where individual sensors exhibit only minimal monotonicity. This method—an instance of equation discovery—searches for functional combinations of sensor with substantially higher monotonicity.

The structure of the paper is as follows. Section 2 provides an overview of the proposed method. Section 3 describes the experimental setup. The results are presented in Section 4 and discussed in Section 5. Finally, Section 6 presents our concluding remarks.

## 2. Method

The goal of our proposed method is to find a proxy for system degradation using available sensor data. As stated earlier, this is a non-trivial task, since the raw sensor data often does not directly capture the degradation of a system. Furthermore, it is hard to verify that a found proxy actually reflects the degradation. In order to validate the proxy, we have formulated three criteria that a potential proxy must meet:

1. Monotonicity
2. Diversity
3. Explainability

These criteria will serve as a benchmark for evaluating potential proxies for system degradation and allow us to say with greater certainty that a proxy actually reflects the health of a system (rather than some external influence).

The first criterion states that a potential proxy must exhibit sufficient monotonicity. System degradation is a process that—barring repairs—always progresses in one direction [19]. If a sensor measurement exhibits a high degree of monotonicity, it is a strong candidate as a proxy for system degradation. By analysing the monotonicity of sensor data, we can identify variables that closely track this unidirectional change. As a way of formalising monotonicity, we propose using the *rank correlation* between the sensor values and their associated time stamps: if one were to sort the sensor values, the degree of order in the associated time stamps is a measure of their monotonicity. There are a number of measures for rank correlation, but in this paper, we will build on the well-known *Spearman’s rank correlation coefficient* [8], which is defined as follows:

(1) $$\rho \left(X,T\right)=\frac{\mathrm{cov}(R(X),R(T))}{\sigma_{R(X)}\sigma_{R(T)}}.$$

where *X* and *T* are the raw scores. In our particular case, *X* denotes the sensor or proxy data, and *T* denotes the timestamp. These raw scores are translated into rank variables using *R*. cov(R(X),R(T)) is the covariance of the rank variables, and $\sigma_{R(X)}$ and $\sigma_{R(T)}$ are the standard deviations of the rank variables.

A proxy with a periodic signal is unlikely to reflect the system’s health, since, as established, the system’s health moves in a single direction. When the individual sensors are not monotonic, constructing a monotonic proxy becomes non-trivial. To address this challenge, we developed the Latent Monotonic Feature Discovery (LMFD) algorithm. LMFD enables us to construct monotonic proxies even when the original sensor inputs are not monotonic. Further details on the LMFD algorithm are provided in Section 2.2.

The second criterion states that a potential proxy is required to have a sufficient degree of diversity in terms of sensors included in the proxy. In other words, a proxy is required to depend on a set of sensors identifiable by common structural properties such as the location, orientation, or method of attachment. Using a single sensor, or all sensors of the same type, as a proxy for degradation can be problematic, because sensors can experience sensor drift or some other type of malfunction as described by [20]. By selecting a *set* of sensors based on structural properties, we reduce the chance of mistakenly interpreting *sensor* drift as system degradation. If the dataset contains individual sensors that already exhibit sufficient monotonicity, we can apply subgroup discovery, explained further in Section 2.1, to identify a subgroup of sensors that are both monotonic and structurally coherent. We then compute the median value across this subgroup, which serves as a robust potential proxy for the health of the system.

The third criterion states that the proxy should be explainable. This implies that the proxy itself should be interpretable (in terms of the sensors involved or their shared structural properties), or should exhibit different rates of change over time that can potentially be linked to environmental factors (such as prolonged periods of frost). To do this, we analyse the first derivative of the proxy and compare it to external data sources such as weather or traffic data. This allows us to compare periods of increased degradation with unusual or extreme conditions and serves as a verification that the produced proxy indeed captures a trend related to the health of the system.

We note that a proxy should adhere to all three requirements for it to be a potential candidate for degradation. But even then, it remains a proxy that requires further interpretation by a domain expert to assess what underlying degradation mechanisms are captured by the monotonic proxy. We argue that, despite some uncertainty about which mechanism the proxy captures, any monotonic behaviour reflects degradation in one way or another.

### 2.1. Subgroup Discovery

Subgroup discovery—a building block in our method—is a branch of machine learning that is concerned with the exploratory analysis of tabular data [12,13,21]. Subgroup discovery (SD) methods aim to uncover statistical correlations between various columns in the data and one specifically identified column, the *target*. The correlations are established by discovering subgroups of rows in the table where the target values show a surprisingly different distribution from that of the entire data. SD considers a search space of subgroups, identified by one or more conditions on the non-target columns (*description attributes*), and reports any subgroups of sufficient interest it finds. For example, in a table of sensors and associated degrees of monotonicity denoted as ρ, it might find a subgroup of sensors identified by certain properties (e.g., “strain gauges oriented longitudinally in area XYZ”) that shows significantly elevated monotonicity. This subgroup indicates that there is a statistical correlation between the description attributes *sensor type*, *sensor orientation*, and *sensor region* on the one hand, and the sensor’s monotonicity on the other. Subgroup descriptions are deliberately relatively concise (compared to the average black-box machine learning method) to allow easy inspection by domain experts.

Subgroup discovery considers an exponentially large search space of potential subgroups based on subsets of description attributes with associated numeric thresholds (e.g., xlocation≤5). In order to restrict the search to a manageable subset of candidate subgroups, often a form of heuristic search is employed to focus the search on more promising regions of the search space. In this paper, we will use beam search [22], and additionally restrict the search to subgroups of modest complexity (up to three attributes at most) to promote interpretability.

Because the technical details of subgroup discovery are outside the scope of this paper, we refer to [12,13] for how SD works. In this paper, we employ the well-established SD toolkit *SubDisc* (https://www.github.com/SubDisc (accessed on 15 January 2026)), formerly known as Cortana [21]. This toolkit comes in the form of an efficient Java implementation with graphical user interface as well as a Python wrapper allowing programmatic SD runs. SubDisc offers a range of SD settings (of which the regression setting [23] is relevant for our application), and is highly parametrisable. A procedure known as *swap randomisation* guarantees that the large space of hypotheses (candidate subgroups) does not lead to spurious findings [24]. It does this by establishing a *distribution of false discoveries* by running SD on randomised versions of the data and then computing a *p*-value from this for the actual findings. SubDisc will be employed for (a) identifying interesting subgroups of sensors showing elevated monotonicity, and (b) identifying periods of increased degradation based on the first derivative of candidate monotonic proxies.

### 2.2. Latent Monotonic Feature Discovery

The goal of our proposed Latent Monotonic Feature Discovery (LMFD) algorithm is to find combinations of individual features that produce a monotonic output. In this work, this directly relates to the task of finding a proxy for the degradation of a system. The LMFD algorithm finds combinations of individual sensor data that produce a monotonic output. This output can then be seen as a latent monotonic feature, hence the name. The basic version of this algorithm, applied to a wide range of application areas (including, but not limited to civil engineering), was first presented by Toussaint et al. [14].

The core algorithm is inspired by other equation discovery approaches such as [25,26]. While these methods have proven useful in the context of regression [18] or classification, we aim to extend the notion of equation discovery to the task of identifying latent monotonic features hidden in the data. By replacing the traditional loss function with the absolute Spearman’s rank correlation, as described previously, we shift the optimisation challenge into the direction of finding monotonic combinations of features.

The proposed algorithm addresses the challenge of searching through a theoretically infinite space of possible equations by combining arithmetic structure selection and parameter optimization in an iterative process. First, candidate equation structures are determined, and then the parameters within these candidate equations are optimized. Based on promising equations, new candidate structures can be generated and evaluated by adding additional terms, repeating the cycle until a satisfactory solution is found.

To make this search feasible, we limit the set of valid equations using a context-free grammar, as is customary in equation discovery [16]. A context-free grammar is defined as follows: G=(N,T,R,S), which contains the following sets of symbols and operations:

- N contains all non-terminal symbols.
- T contains all terminal symbols.
- R contains the rewrite rules in the form A→α where A∈N and $\alpha \in {\left(N\cup T\right)}^{*}$.
- S contains the start symbols.

The grammar defines which equations can be constructed, and by applying its rewrite rules (R) step by step, we obtain a top-down search strategy for exploring possible structures. This framework naturally supports the use of beam search [22], which allows for the exploration of multiple promising equation structures at each step (reminiscent of how beam search is used in subgroup discovery). Instead of keeping only the single best candidate, beam search maintains a set of strong candidates considered so far, with the *beam width* determining how broadly the algorithm explores the space of possible equations. By allowing the algorithm to not only select the best candidate at each level, but keeping a list of promising ones, the algorithm can avoid local minima, allowing the algorithm to find deep structure.

Given a candidate equation, the next step is to optimise its associated parameters. Parameter optimisation is a non-trivial but widely studied problem. A central difficulty in our setting is that optimisation with respect to rank correlation is not amenable to gradient-based methods, which form the dominant paradigm for optimising complex functions. For this reason, we employ the Nelder–Mead algorithm [27], which enables optimisation of the non-differentiable objective functions encountered here.

For the task of discovering latent monotonic features, we have constructed the following grammar *G*:

- N={V,B,X}
- $T=\{s_{1},s_{2},\ldots ,c,+,\cdot ,\exp\}$
- R={V→A|V+B,
      A→X|exp(c·X),
      B→c·X|c·exp(c·X),
  $\;\;\;\;X\to s_{1}\;\left|\;s_{2}\right|\;\ldots$ }
- S={V}.

This grammar produces equations of arbitrary length, consisting of a series of summands (additive terms) involving either a term $c_{1}s_{i}$ (linear in the sensor $s_{i}$) or a term $c_{2}\cdot \exp\left(c_{3}s_{i}\right)$ (exponential). Note that while *G* only considers addition, subtraction is implicitly provided for by allowing *c* to be negative. Parameters $c_{1}$ and $c_{2}$ weigh the contribution of the term within the equation, whereas $c_{3}$ ‘stretches’ the sensor values before applying the exp function. The algorithm is allowed to mix linear and exponential terms as it sees fit. The purpose of the exponential terms is to allow the sensor values to be mapped to the [0,∞] range, with larger values being emphasised and smaller values being suppressed. Whether this transformation is required in our domain remains to be seen, but it is customary in equation discovery to add one or more non-linear terms.

From a search point of view, the set of equations generated by grammar *G* is traversed top-down. The first set of equations considered is simply $s_{1},\ldots ,s_{n}$: the individual sensors. Note that the single terms in these equations do not have a parameter *c*, since from a monotonicity perspective, it has no effect (multiplying any function by a constant does not change its degree of monotonicity). At depth d=2, promising equations from the first level (in terms of |ρ|) are extended with a second summand—one that does have a parameter *c* now, since the two terms need to be relatively weighed, e.g.,

(2) $$s_{i}+cs_{j}$$

The top-down additive way of building equations is attractive, since candidate equations are functionally similar to their ‘offspring’, increasing the chances of promising candidates leading to even more monotonic equations. To restrict the search space of potential equations to a manageable size, both in terms of computation and interpretability of the equations, we introduce a maximum search depth $d_{max}$, which reflects the summands in a single equation.

## 3. Experimental Setup

In this section, we describe the experimental setup for our work. The experiments are divided into three parts as follows: First, we look at individual sensors and treat an individual sensor as a proxy for the health of a system. Second, we perform subgroup discovery and find a subgroup of sensors that is significantly more monotonic than the average of all sensors. We then look at this subgroup as a proxy for the health of the system. Finally, in the third experiment, we test our LMFD algorithm to construct a monotonic proxy from non-monotonic sensors by holding out overly monotonic sensors from the dataset and searching for combinations of sensors that provide equally monotonic proxies.

### 3.1. Data Description

**Figure 1:**
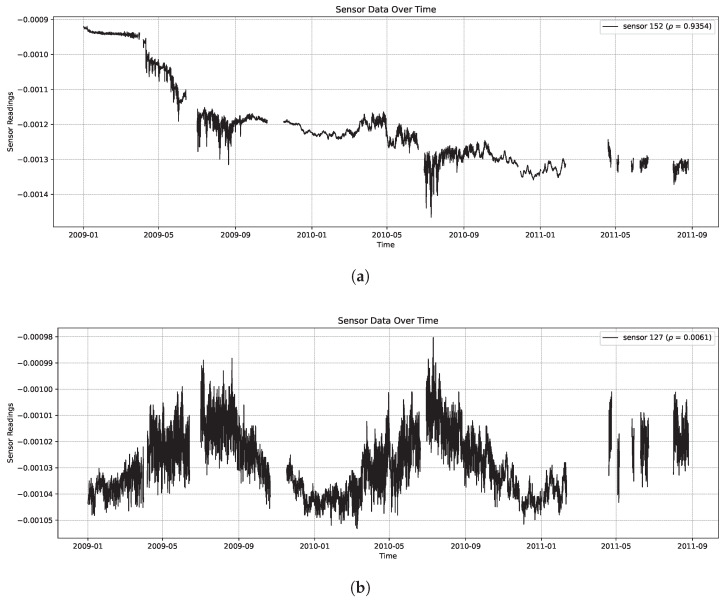
Subfigure (**a**) shows a sensor with reasonably monotonic behavior (|ρ|=0.935), while subfigure (**b**) shows a sensor with very little monotonicity (|ρ|=0.006). The sensor in subfigure (**b**) is highly sensitive to seasonal influences (the data covers two years) as well as daily fluctuations caused by weather and traffic.

Our experiments revolve around a dataset obtained from the InfraWatch project [28,29]. This dataset includes hourly measurements from 119 sensors over a time frame of roughly two years, resulting in 17,996 data points. Figure 1 shows examples of sensor data for two sensors. Note that there are some periods where no data was collected, due to maintenance or power outages. Since we are mainly interested in the rank correlation, the missing data is not an issue for our task. These sensors are of three different types (strain gauges, geophone, and temperature) and are distributed over multiple locations on the *Hollandse Brug*, a Dutch concrete highway bridge.

**Figure 2:**
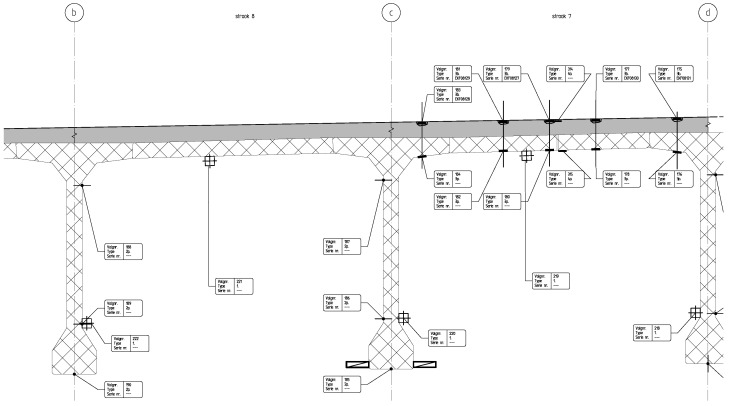
A cropped version of the sensor placement blueprint. The blueprint is an intersection of the bridge showing the first three girders, denoted as *b*, *c*, and *d*. Furthermore, on the lane denoted as ‘strook 7’, the placement of the road surface sensors can be seen.

In order to be able to group sensors (as per our method aimed at redundancy), additional information about the sensors is required. To achieve this, we have created a meta-features dataset which contains meta-information for each sensor about the location, orientation, and type of the sensor. Figure 2 illustrates the sensor placement for a part of the bridge (about 20%). The figure represents a part of the cross-section of the bridge. The meta-features are as follows:

- *Sensor ID:* The ID assigned to the sensor.
- *Sensor Type:* The type of the sensor. This can be one of the following: strain gauge, geophone, or temperature.
- *Location X:* The location of the sensor in longitudinal direction.
- *Location Y:* The location of the sensor in transverse direction.
- *Orientation:* The orientation of the sensor. For strain gauges, this can be either in the longitudinal or transverse direction. For geophones this can be in the vertical direction. For temperature sensors, this can either be an indication that the sensor is located within the concrete structure or that it is attached to the concrete structure.
- *Location G/B:* This feature denotes on which girder (G) or band (B) the sensor is placed. The first three girders are shown in Figure 2, and are denoted with *b*, *c*, and *d*.
  The first two bands are shown in Figure 2, and are denoted with *strook 0* and *strook 1*.
- *Location:* This feature denotes where on the girder or band the sensor is placed.
- *|ρ|:* This feature shows the absolute Spearman’s rho value for the sensor, calculated as the absolute value of Equation (1).

In order to correlate periods of high degradation with atmospheric conditions, additional data sources were collected. In this work, we use weather data from the Dutch meteorological institute KNMI (Koninklijk Nederlands Meteorologisch Instituut, https://www.knmi.nl/nederland-nu/klimatologie/uurgegevens (accessed 15 January 2026)). The closest weather station available is Lelystad and is located around 20 km from the bridge. It is important to note that the weather station is situated at a considerable distance from the bridge. While this distance is unlikely to impact the assessment of general weather patterns, it may influence the accuracy of measurements related to more localized weather phenomena. Therefore, the station’s location should be considered when interpreting results involving local weather effects. The dataset includes measurements of temperature, sunlight, cloud cover, visibility, air pressure, wind, and precipitation.

### 3.2. Details of Experimental Setup

Our first experiment starts off simply by computing the monotonicity of each sensor by computing the absolute Spearman’s rank correlation for each sensor individually over the time series of 17,996 individual measurements. We rank the sensors according to resulting monotonicity |ρ| and report the top and bottom five sensors, representing the most monotonic and most periodic/intermittent sensors, respectively. The top and bottom of the ranking are compared to the average monotonicity computed as the average |ρ| over 119 sensors.

The analysis proceeds with a subgroup discovery exercise based on the sensor meta-features and the computed monotonicities. The dataset produced here consists of 8 features (including |ρ|) and 119 rows. The SD analysis was executed using SubDisc, selecting the *Single Numeric* option, which indicates a regression setting [30]. The target was |ρ| and the quality measure of choice was z-score, a common quality measure in SD. z-score is a measure of deviation of the target values within a candidate subgroup, computed by comparing the average target value within the subgroup to the population average, dividing by the standard deviation of the population (for normalisation), and scaling this product by the square root of the size of the subgroup. This setting encourages the discovery of subgroups of at least moderate size that show a surprisingly high monotonicity.

The actual run was started with the following settings (for reproducibility purposes): beam search was used with a beam width of 100 (candidates per level), and a maximum depth of 3 (conditions). Numeric thresholds were determined using the ‘best’ option, without binning. Subgroups were required to contain at least 8 sensors. The minimum subgroup quality (in terms of *z*-score) was determined using the swap randomisation procedure to be $Q_{min}=3.27$. Only subgroups exceeding this level will be reported as significant, at a significance level of α=0.05. We will report the sensors within each subgroup, analyse the meta-features that define the subgroup descriptions, and create a proxy based on the median of the identified sensors.

The next step is to connect the first derivative of this proxy to the meteorological data. This will produce a secondary dataset that measures 21 wide and 17,971 long. The features consist of 19 atmospheric indicators plus a timestamp and the target (the first derivative, identified as δ). The number of rows corresponds to the number of time points in the original sensor data (17,996) for which a measurement exists that is one hour in the past (to obtain an estimate of the first derivative). The difference between the two counts thus indicates the number of gaps: 17,996 − 17,971 = 25.

The parameters of the secondary SD run were mostly identical to those of the first. We list the changes. Instead of z-score, we used the inverse *z*-score, which is simply the *z*-score negated. This guarantees that the algorithm searches for subsets of atmospheric conditions that identify surprisingly *low* values of the target (negative values indicate a drop in the proxy). The minimum subgroup size was set to 500 (time points), which corresponds to roughly 2.8%. We ran the swap randomization procedure to compute the minimum threshold for qualities, which turned out to be $Q_{min}=5.782$ at a significance level of α=0.05.

Finally, we test the ability of the proposed LMFD method to discover monotonic proxies in the absence of sensors with monotonic tendencies. As the described InfraWatch dataset already contains clear monotonic sensors, we create a challenge for LMFD by producing extracts from the data in two ways. First, we use the reported subgroup from the previous experiment, which lists a number of clearly monotonic features, and remove these sensors from the dataset. This leads to the first derived dataset of 109 sensors. A second—more challenging—derived dataset is produced by removing all sensors with |ρ|≥0.3, which results in a dataset of 56 sensors.

Parameter settings for LMFD were as follows. For beam search, a relatively modest beam width of w=10, and a search depth of $d_{max}=3$ results in a search that is sufficiently extensive while not requiring excessive computation (in the order of 30 min).

The LMFD run should result in a ranking of the most interesting discovered equations, of which we will report the most monotonic proxy. It should be noted that these proxies consist of multiple sensors, which means that we cannot derive a secondary dataset based on the sensor meta-features, as we do in the second experiment (based on individual sensors). As a corollary, we cannot group proxies in order to prove that individual proxies do not reveal sensor drift. Also, simply taking the top 10 proxies may not work, because they could all be variations on a theme. For example, the proxies may be composed of one or two central sensors (that could contain a sensor drift), combined with arbitrary sensors with a tiny constant. To demonstrate redundancy in the proxies under these limitations, we will take an iterative approach: after finding an optimal proxy, we remove the sensors involved from the (already reduced) dataset, and repeat the search for monotonic proxies. If multiple disjoint combinations of sensors can be found in this iterative manner, this demonstrates that the proxies don’t rely on individual degrading sensors.

A valid question in this context is whether the found proxies are significant findings, or perhaps spurious findings resulting from random effects in the data. In our search process, we are considering a considerable number of candidate proxies and adapting our search to intermediate promising results, so that even on random data, this process may produce spurious proxies with a monotonicity of |ρ|>0. In order to judge whether our results on actual data are significant findings (i.e., not due to chance), we run a Monte Carlo process based on swap-randomised versions of the data at hand, and also run LMFD on these randomised datasets. A swap-randomised dataset is produced simply by taking the original dataset and replacing the timestamps with a random permutation of those timestamps. This breaks the relationship between the sensor data and the timestamp, while retaining the distribution of sensor values and the relationships between sensors. The Monte Carlo process on 100 swap-randomised datasets will produce 100 results (taking the |ρ| of the best proxy found in each run) from which we can compute a ‘distribution of false discoveries’, which can then be compared to the |ρ| of the actual run.

## 4. Experimental Results

In the following three sections, we describe our three main experiments. Section 4.1 presents the results for individual sensors. Section 4.2 examines how naturally monotonic sensors can be grouped to construct a robust aggregated proxy that can be correlated with meteorological data. Finally, Section 4.3 reports the results of applying LMFD to sensors that are not naturally monotonic.

### 4.1. Individual Sensors

**Figure 3:**
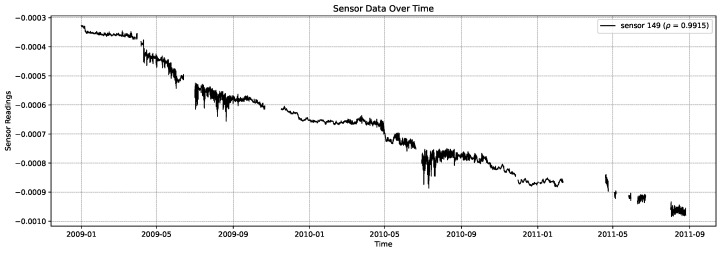
Sensor data over time of the most monotonic sensor in the dataset.

Over the 119 sensors considered in the InfraWatch data, the average monotonicity (absolute Spearman’s rank correlation) was found to be |ρ|=0.406, showing a moderate trend over the two years of data involved (‘moderate’ being the generally accepted qualitative term for correlations between 0.4 and 0.6). But amongst the sensors, there is a huge variance in monotonicity, ranging from |ρ|=0.006 for the most periodic, least monotonic sensor $s_{127}$ (shown at the bottom of Figure 1), to the most monotonic sensor $s_{149}$ with |ρ|=0.992 (shown in Figure 3). This last sensor concerns a cast-in strain gauge that happens to be oriented longitudinally on top of the bridge. The least monotonic sensor $s_{127}$ also concerns a cast-in strain gauge, oriented transversely, in the road surface. This sensor is highly periodic, showing considerable correlation with the outside temperature. The top five of most monotonic sensors is listed in Table 1, along with their meta-features.

It thus appears that this dataset has the fortunate presence of highly monotonic sensors (as well as some highly intermittent or periodic sensors). This is certainly not the case for all SHM datasets, so the results obtained here cannot be thought of as typical for SHM. But still, as described in earlier sections, it might be the case that sensor $s_{149}$ is showing a monotonic trend as a result of local drift in the sensor itself, so that it cannot immediately be used as a reliable proxy for ageing or degradation. We need to further examine the list of sensors, their meta-features and resulting monotonicities, to see if subgroups of sensors can be identified that *do* suggest reliable proxies for degradation, while also providing insight into the specific common characteristics that other—less monotonic—sensors do not have.

### 4.2. Subgroups of Sensors

Subgroup discovery over the collection of 119 sensors, with |ρ| as the target revealed an interesting subgroup of 10 sensors. This subgroup has a z-score of z=5.11, far exceeding the minimum of $Q_{min}=3.27$. Its description is as follows:

(3) location=‘top’∧x≥7,

meaning a group of sensors located at the top of the bridge and positioned at larger *x* coordinates (7 and 9, to be precise). Furthermore, these sensors have the following in common:

- They are strain gauges.
- They are cast-in.
- They are placed in band 4 or 5.

The orientation within this subgroup can be both longitudinal and transverse.

**Figure 4:**
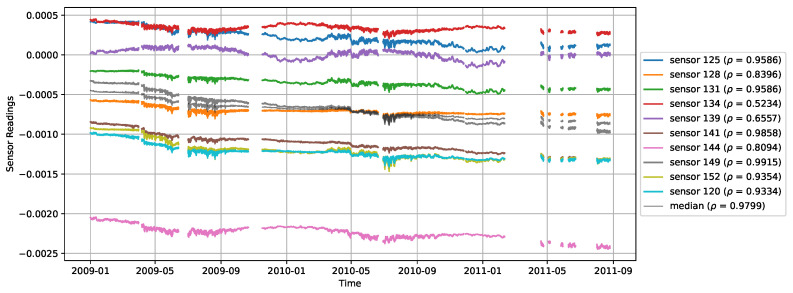
A subgroup of 10 monotonic sensors and its derived median proxy (in black).

The average monotonicity of this subgroup is |ρ|=0.859, with individual values ranging from 0.523 ($s_{134}$) to 0.992 ($s_{149}$). This subgroup identifies a set of sensors with specific features that have to do with structural properties (rather than sensor-related), so we have to conclude that this collection of sensors provides a reliable proxy for health. If the group of sensors would be sensor-type-related, the observed phenomenon would rather be caused by sensor drift, which can be ruled out here. Figure 4 shows the progression of sensor values over time, for the 10 sensors involved, and also for the smoothed median sensor, which has a monotonicity of |ρ|=0.985. Note that this composite proxy may not be as monotonic as the best-performing sensors, but is more robust against the future failure of individual sensors.

**Figure 5:**
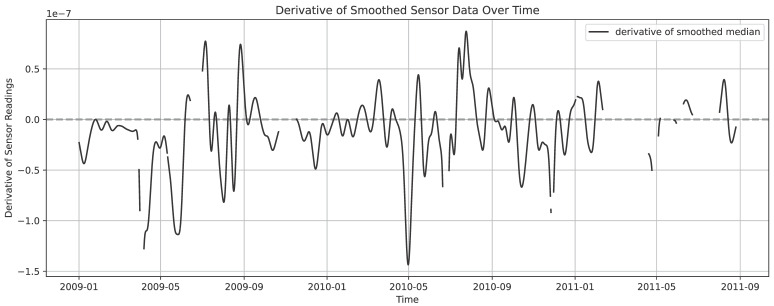
The first derivative of the median shown in Figure 4. Gaussian smoothing is applied for presentation purposes.

To better understand the reasons behind degradation captured by this subgroup of 10 sensors, and to analyse the circumstances under which this progress is most prominent, we continue by relating the first derivative of the proxy to meteorological conditions, as per our proposed methodology. This first derivative is presented in Figure 5.

Subgroup discovery on the joint meteo/degradation data revealed one prominent subgroup, identified by the following conditions:

(4) Relativehumidity≤59%∧Temperature≥17.3°∧Globalradiation≥51.

This subgroup captures 5.2% of the cases. The average degradation during such circumstances is $\delta =-4.386\times 10^{-6}$, compared to $\delta =-1.583\times 10^{-8}$ for the entire dataset. For the complement—all cases not covered by the provided conditions—the degradation is actually slightly positive: $\delta =2.219\times 10^{-7}$.

**Figure 6:**
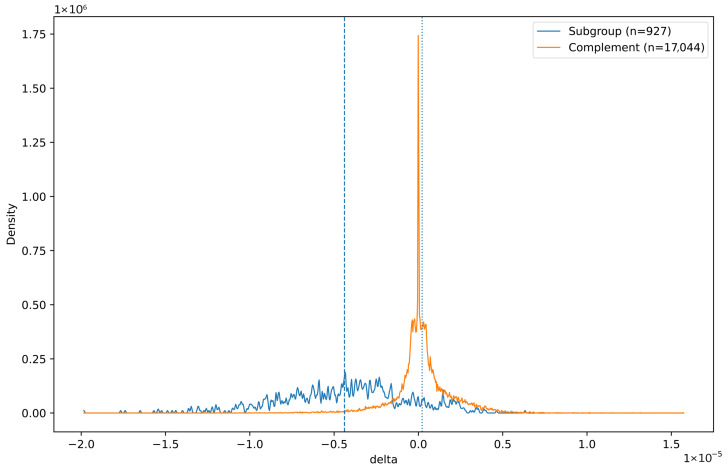
Subgroup capturing substantial periods of degradation. The values along the x-axis represent the difference between consecutive data points, i.e., the degradation. The blue curve represents the probability density function of the subgroup (representing 5.2% of cases), showing largely negative values. The distribution of the remaining sensors is presented as the orange curve, which is nearly centred around 0. Vertical dashed lines indicate the respective averages.

Figure 6 presents the δ distribution of the subgroup (blue) compared to that of the complement (orange) as computed by kernel density estimation. The δ is computed as the difference between consecutive data points, thus approximating the first-order derivative of the proxy. Note that this figure does not correctly represent the relative sizes of the two subgroups: the complement is roughly twenty times larger than the subgroup. Clearly, subgroup δ values are substantially lower than those of the complement, which are nearly centred around 0. In order to test for the significance of this subgroup, we ran the swap randomization procedure to obtain a DFD and a minimum threshold for qualities (in terms of negative *z*-score here), which turned out to be $Q_{min}=5.782$ at a significance level of α=0.05. The actual quality of our subgroup is substantially bigger: z=63.46, making our finding highly significant. The associated *p*-value confirms this: p<0.00001.

### 4.3. Latent Monotonic Feature Discovery

In order to emulate a more challenging scenario, where no monotonic sensors are present in the data, we perform the following. First, we take the monotonic subgroup from (3) and remove all 10 sensors involved from our dataset. Then, with the remaining sensors, we perform LMFD, resulting in the following proxy:

(5) $$s_{165}-0.9322\cdot s_{168}+0.1847\cdot s_{197}$$

**Figure 7:**
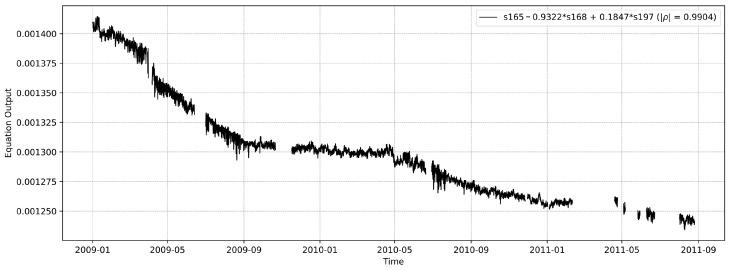
The proxy as described by Equation (5). The plot shows its value over time.

This proxy has an absolute Spearman’s rank correlation of |ρ|=0.990, which outperforms the individual sensors it is derived from, as Table 2 demonstrates. Figure 7 presents the associated proxy.

In order to show that the reported combination of sensors is not the only possible proxy, and other combinations achieve similar results, we repeat the LMFD exercise with sensors $s_{165},s_{168},$ and $s_{197}$ removed (on top of the initial 10 removed sensors). This results in the following proxy with a |ρ|=0.979 (only marginally lower):

(6) $$s_{108}-0.2342\cdot s_{195}+0.1504\cdot s_{236}$$

We note that while the subgroup (3) removes the most monotonic sensors from our dataset, some reasonably monotonic sensors still remain, notably $s_{197}$ used in (5). One could argue that this sensor, with an absolute Spearman’s rank correlation of |ρ|=0.957 but outside the subgroup of 10 sensors, is still unrealistically monotonic for an average SHM scenario. To evaluate our proposed system in a case where all sensors can be classified as periodic, we have removed all sensors where the degree of monotonicity is greater than 0.3, and ran LMFD again and found a proxy with |ρ|=0.957. Equation (7) shows the equation for the found proxy.

(7) $$s_{159}-0.9443\cdot s_{165}-0.0413\cdot s_{238}$$

The monotonicity values for the involved sensors, compared to the resulting proxy, are given in Table 3.

Again, we show the proxy closest in performance that does not include any of the previously used sensors, with an |ρ|=0.932, and the following shape:

(8) $$s_{119}-1.1741\cdot s_{124}-0.3950\cdot s_{176}$$

**Figure 8:**
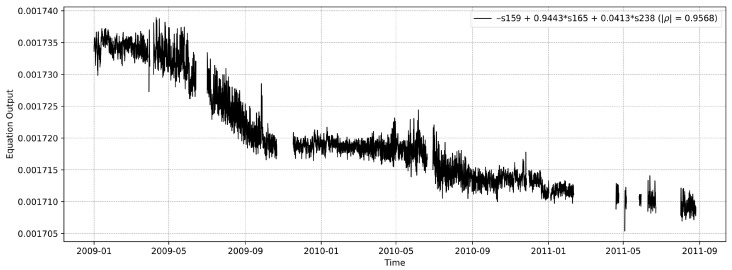
Plots of the equation found with the highest degree of monotonicity. All individual sensors with a |ρ|>0.3 have been removed to make the task more challenging. Note the similarity with Figure 7.

Figure 8 shows the proxy as a function of time. Interestingly, we observe that the general shape aligns with the previously found proxy, indicating that both proxies seem to identify similar time periods as periods of increased degradation.

**Figure 9:**
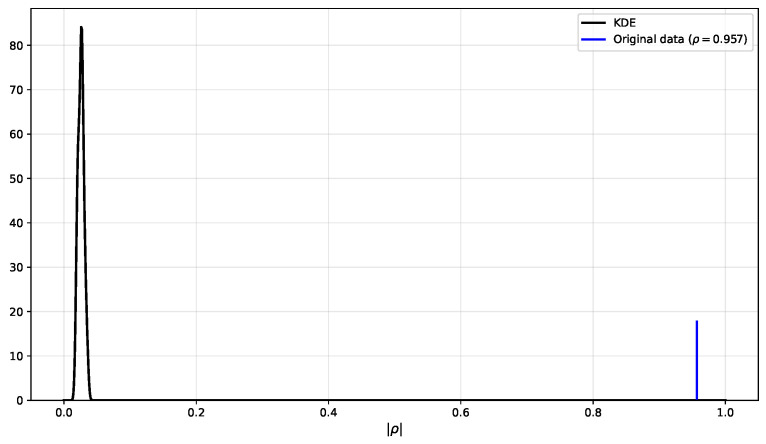
Distribution of the results on 100 swap-randomised datasets (left bell-curve), compared to the actual results (blue peak on the right). The obtained result is clearly not produced by random effects in the data.

To confirm that this is a valid finding, and not caused by random effects or extensive testing of candidate proxies, we applied the Monte Carlo experiment with 100 swap-randomised versions of the data (as announced in Section 3). The results of this can be found in Figure 9. Clearly, the results on random data are situated in the range of |ρ|∈[0.017,0.037] (mean 0.026), compared to |ρ|=0.957 for the actual results. It is safe to conclude that the found proxy is not due to random effects or excessive multiple hypothesis testing.

## 5. Discussion

**Figure 10:**
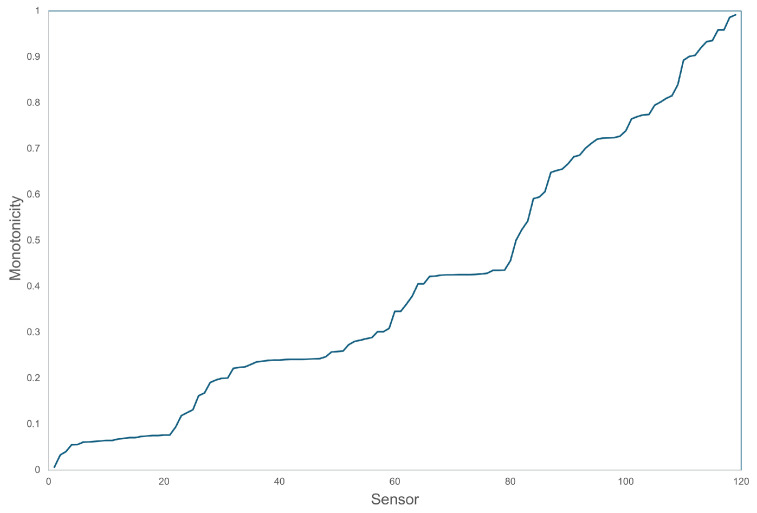
Monotonicity of the InfraWatch sensors, from non-monotonic (periodic or intermittent) on the left, to highly monotonic on the right.

In our experiments, we were able to unearth a number of proxies for degradation, either as individual sensors or as combinations of sensors found through extensive search of the LMFD algorithm. We should acknowledge that we were fortunate to ‘stumble upon’ multiple monotonic sensors, which is certainly not typical for SHM applications. In most monitoring datasets, the measured signal is dominated by dynamic influences—such as weather and traffic—that are unrelated to structural ageing, making degradation proxies considerably harder to identify. Although the InfraWatch setup is fortunate, we would like to stress that the degree of monotonicity of the sensors ranges from very present to completely absent. Figure 10 shows the distribution of |ρ| for the sensors involved.

The sensor setup on the Hollandse Brug was designed to be highly redundant, with a wide variety of sensors in terms of sensor type, placement, attachment, and orientation. This over-designed setup was selected for exactly the goals presented in this paper: to better understand what sensor configuration would provide the best proxies for health, both for single-sensor placement, as well as for combinations of sensors. On different structures with a less extensive (and costly) setup, it is unlikely that single sensors will be monotonic, although combinations of sensors may prove effective, as our LMFD experiments demonstrate.

The experiments related to the group of 10 individually monotonic sensors revealed an interesting correlation between atmospheric conditions and the rate of change of the median proxy. Under circumstances of reduced humidity and increased temperatures and solar irradiation (Equation (4)), a significant reduction in the proxy was observed (see Figure 6). Note that in the temperate maritime climate of the Netherlands (Köppen–Geiger classification: Cfb [31]), these conditions are relatively rare. Although the exact degradation mechanism underlying this intermittent behaviour remains speculative, several forms of concrete deterioration have been associated with concrete desiccation [32,33]. These sources demonstrate that prolonged exposure to low ambient humidity can cause micro-cracking of the concrete. This micro-cracking is a result of tensile stresses in the material due to non-uniform shrinkage in the material. This known phenomenon might explain the statistical pattern observed here, although further research will need to confirm this hypothesis since no material inspection of the highway bridge was conducted.

It is good to stress that the presented proxy (Figure 4) identifies episodes of increased degradation (which can be linked to environmental factors), and these changes are irreversible. Outside these episodes, the structure does not return to the original state, and the proxy shows very little progress. In this sense, it is different from the more usual periodic sensors, which may also have episodes of ‘degradation’, but these are interspersed by episodes of ‘recuperation’. Clearly, such alternative sensors are not good proxies for health.

## 6. Conclusions

This paper investigated how long-term sensor data can be used to derive meaningful proxies for structural ageing in the presence of dominant environmental and operational effects. By adopting monotonicity as a guiding principle, we introduced a framework for identifying indirect yet interpretable indicators of degradation from structural health monitoring data. Two complementary approaches were explored. First, the degree of monotonicity for each sensor was established using Spearman’s rank correlation, after which subgroup discovery identified structurally coherent groups of sensors with elevated monotonic behaviour, enabling the construction of robust aggregated proxies to reduce sensitivity to individual sensor drift. Second, we demonstrated that a novel method called Latent Monotonic Feature Discovery (LMFD) can uncover highly monotonic composite features even when individual sensors are predominantly periodic or intermittent.

Applied to a two-year hourly monitoring dataset of a concrete highway bridge, the proposed methods revealed consistent long-term trends and enabled the identification of periods of accelerated degradation associated with specific environmental conditions (relatively warm and dry, unusual for the local temperate maritime climate). We note that no visual inspection was conducted so no clear evidence of the proposed physical damage was collected. Although the resulting proxies do not measure physical damage directly, they provide a principled, data-driven means of tracking ageing processes over extended time horizons.

Together, these results demonstrate that long-term infrastructure ageing can be modelled indirectly from dense sensing data, even when degradation signals are weak and heavily obscured. Despite the available dataset being rich and varied, which improves the likelihood of encountering naturally monotonic sensors, we demonstrated LMFD’s ability to unearth relatively concise and interpretable proxies for health when obviously monotonic sensors were removed. This demonstrates that LMFD can be employed in more typical SHM datasets with fewer sensors that are dominated by operational and environmental influences.

## Figures and Tables

**Table 1:** Table showing the top five most monotonic sensors in the InfraWatch dataset.

Sensor ID	Sensor Type	Orientation	Band	Location	|ρ|
149	Strain gauge	Longitudinal	5	Top side	0.992
141	Strain gauge	Longitudinal	5	Top side	0.986
131	Strain gauge	Longitudinal	4	Top side	0.959
125	Strain gauge	Longitudinal	4	Top side	0.959
152	Strain gauge	transverse	5	Top side	0.935

**Table 2:** Table showing the degree of monotonicity of the found proxy alongside the individual sensors.

Sensor ID	|ρ|	Sensor Type
165	0.093	cast-in strain gauge, top, longitudinal
168	0.379	cast-in strain gauge, top, transverse
197	0.903	cast-in strain gauge, road surface, longitudinal
LMFD-proxy	0.990	composite

**Table 3:** Table showing the degree of monotonicity of the found proxy of Equation (7) alongside the individual sensors.

Sensor ID	|ρ|	Sensor Type
159	0.132	cast-in strain gauge, top, longitudinal
165	0.094	cast-in strain gauge, top, longitudinal
238	0.246	geophone, deck, vertical
LMFD-proxy	0.957	composite

## Data Availability

The original data presented in the study are openly available from https://github.com/GuusToussaint/data-driven-shm, accessed on 15 January 2026.
